# Disulfidptosis in tumor progression

**DOI:** 10.1038/s41420-025-02495-9

**Published:** 2025-04-28

**Authors:** Senlin Wan, Changming Liang, Chengwei Wu, Song Wang, Jiawei Wang, Lishuai Xu, Xu Zhang, Yinfen Hou, Yabin Xia, Li Xu, Xiaoxu Huang

**Affiliations:** 1 Department of Gastrointestinal Surgery, The First Affiliated Hospital, Yijishan Hospital of Wannan Medical College, Wuhu, Anhui China; 2 Anhui Province Key Laboratory of Non-coding RNA Basic and Clinical Transformation, Wuhu, Anhui China

**Keywords:** Cancer, Cell biology

## Abstract

Disulfidptosis, a regulated cell death modality driven by the cystine transporter solute carrier family 7 member 11 (SLC7A11), is characterized by actin cytoskeleton collapse under glucose starvation. This review systematically elucidates the pivotal role of disulfidptosis in tumor metabolic reprogramming, with a focus on its molecular mechanisms and distinctions from other cell death pathways. The core mechanisms include SLC7A11-mediated cystine overload and NRF2/c-Myc-regulated pentose phosphate pathway activation. By integrating multiomics data and single-cell transcriptomics, we comprehensively decipher the heterogeneous expression patterns of disulfidptosis-related genes (DRGs) and their dynamic interplay with immune microenvironment remodeling. Furthermore, the coexpression networks of DRGs and disulfidptosis-related long noncoding RNAs (DRLs) offer novel insights into tumor diagnosis, prognosis, and targeted therapy. Therapeutically, SLC7A11 inhibitors (e.g., HG106) and glucose transporter inhibitors (e.g., BAY-876) demonstrate efficacy by exploiting metabolic vulnerabilities, whereas natural compounds synergizing with immune checkpoint blockade provide strategies to counteract immunosuppressive microenvironments. Through interdisciplinary collaboration and clinical translation, disulfidptosis research holds transformative potential in redefining precision oncology.

## Facts

- Disulfidptosis is a regulated cell death driven by SLC7A11-mediated cystine overload and glucose starvation, characterized by disulfide stress-induced actin cytoskeleton collapse.
- Disulfidptosis differs significantly from other cell death pathways such as ferroptosis, cuproptosis, and apoptosis, and exhibits intricate crosstalk with them through shared molecular mechanisms and metabolic dependencies.
- Therapeutically, SLC7A11 inhibitors and glucose transporter inhibitors demonstrate efficacy by exploiting metabolic vulnerabilities in tumor cells, while natural compounds combined with immune checkpoint blockade provide strategies to counteract immunosuppressive microenvironments.

## Open questions

- In different types of tumors, how exactly do the heterogeneous expression patterns of disulfidptosis-related genes (DRGs) and their dynamic interactions with immune microenvironment remodeling affect tumor occurrence and progression?
- What are the synergistic mechanisms between disulfidptosis and other types of programmed cell death, and how can metabolic reprogramming be leveraged to optimize combination therapies?
- When using combination therapies targeting disulfidptosis (e.g., SLC7A11 inhibitors combined with immune checkpoint inhibitors), how can we optimize treatment strategies to maximize therapeutic effects while minimizing potential side effects?

## Introduction

Sulfur, as a macroelement, is ubiquitous in nature and accounts for approximately 0.05–0.1% of the Earth’s crust [1]. Disulfides are relatively stable substances that maintain protein stability through inter- and intrasubunit disulfide bonds. The disulfide bond formed between the thiol groups in the two cysteine (Cys) residues is an important part of the secondary and tertiary structure of proteins, and this bond plays a significant role in maintaining the stability of the protein steric structure [2]. Disulfidptosis is caused by disulfide stress due to the abnormal accumulation of intracellular disulfides such as cystine, which disrupts the balance of the cellular redox state and thus is highly toxic to the cell [3]. Nicotinamide adenine dinucleotide phosphate (NADPH) is a reducing agent that reduces disulfides, thereby maintaining cell survival and protecting cells from damage. NADPH in the cytoplasm is produced primarily from glucose via the pentose phosphate pathway. High expression of the cystine transporter solute carrier family 7 member 11 (SLC7A11; also known as xCT) in tumor cells leads to increased cystine uptake and the reduction of cystine to Cys, which consumes a large amount of NADPH and leads to the accumulation of disulfides in the cell. Moreover, if glucose deficiency occurs, the inability to rapidly produce NADPH allows for the accumulation of high levels of intracellular disulfides, leading to rapid cell death [4]. This type of cell death associated with disulfide stress is called disulfidptosis [5].

Maintaining redox balance is essential for cell survival [6]. Genetic mutations and metabolic reprogramming cause greater oxidative stress in tumor cells than in nontumor cells [7, 8]. For tumor cells to survive and proliferate, they must maintain sufficient levels of glutathione (GSH) to counteract excessive intracellular reactive oxygen species (ROS). GSH is a tripeptide of glycine, Cys, and glutamic acid that is proposed to be used as a storage form of Cys [9]. Most tumor cells in vivo rely primarily on SLC7A11 to translocate extracellular cystine into the cytoplasm and reduce it to Cys in the presence of NADPH. Therefore, SLC7A11 is considered a key gene for maintaining tumor cell survival and tumor cell antioxidant defense [10]. However, the recent discovery of disulfidptosis led us to understand that SLC7A11 can induce cell death under glucose deprivation conditions and that this new form of regulated cell death (RCD) [11] is different from other forms of cell death, revealing a new frontier of RCD and a new mechanism by which organisms can combat malignant tumors. As other types of RCD can be exploited for cancer treatment, disulfidptosis-related treatments could represent a new direction in tumor therapy.

## The occurrence of disulfidptosis

### A brief description of sulfur metabolism

**Fig. 1:**
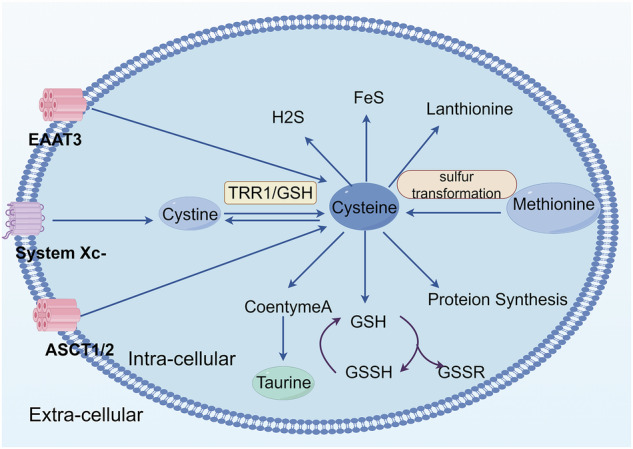
Basic S metabolism. Cysteine is imported through the excitatory amino acid transporter 3 (EAAT3) and the alanine, serine, and cysteine transporter (ASCT). Its oxidized form cystine is transported by the transporter system xc^−^. Once inside the cell, cystine is rapidly reduced to cysteine by thioredoxin reductase 1 (TRR1) or glutathione (GSH). Cysteine is consumed via various metabolic pathways such as protein synthesis and the production of sulfur-containing molecules such as glutathione, taurine, lanolinothioneine, coenzyme A, and the gaseous signaling molecule hydrogen sulfide (H2S). Methionine is converted to cysteine via sulfur transformation. This figure was created with Figdraw.

Sulfur is a core element involved in maintaining redox homeostasis and energy metabolism in mammals, and its metabolic network involves the coordinated regulation of the methionine (Met) cycle, trans-sulfuration pathway, and GSH system. Sulfur is primarily derived from dietary sulfur-containing amino acids—Cys and Met—which exist in the forms of thiol (-SH) and thioether (-S-), respectively—and play central roles in various metabolic pathways. Met, an essential amino acid, cannot be synthesized de novo and must be obtained from dietary protein [12, 13]. Met serves not only as the initiator amino acid for protein synthesis but also as a methyl donor (e.g., S-adenosylmethionine, SAM) through the Met cycle, participating in critical processes such as DNA methylation and polyamine synthesis [14, 15]. Cys can be synthesized from Met via the trans-sulfuration pathway. The intake form of Cys is often oxidized cystine (Cys-S-S-Cys), which enters the cytoplasm through membrane transporters (e.g., SLC7A11/xCT) and is reduced to free Cys by NADPH-dependent disulfide reductase systems [12, 16, 17] (Fig. 1).

The Met cycle involves the stepwise conversion of Met to SAM, S-adenosylhomocysteine, and homocysteine (Hcy), which are further metabolized through remethylation or the trans-sulfuration pathway [18]. The trans-sulfuration pathway transfers the sulfur atom from Hcy to serine, generating Cys while releasing α-ketobutyrate and ammonia. Cys acts as a metabolic hub in cells: Cys is a key precursor for GSH (γ-L-glutamyl-L-cysteinylglycine) [19]. As a major antioxidant, GSH scavenges ROS through the GSH/GSSG cycle and plays a role in drug detoxification. Additionally, Cys participates in the biosynthesis of coenzyme A (CoA), iron‒sulfur clusters (Fe‒S), taurine, and phosphoadenosine phosphosulfate (APS/PAPS), which are indispensable for energy metabolism, signal transduction, and sulfation reactions [20, 21].

The unique redox versatility of sulfur makes it a central player in oxidation‒reduction reactions. Glutathione peroxidases (GPXs) and peroxiredoxins rely on GSH or thioredoxin systems to eliminate ROS such as H₂O₂, maintaining the intracellular redox balance [22–24]. Furthermore, methionine sulfoxide can be reduced back to Met by methionine sulfoxide reductases, repairing oxidized proteins and potentially participating in oxidative stress signaling regulation [25]. Dysregulation of sulfur metabolism is closely linked to the pathogenesis of various diseases, involving imbalances in redox homeostasis, disrupted methyl donor metabolism, and defects in the synthesis of sulfur-containing molecules. In neurodegenerative diseases, GSH depletion leads to mitochondrial complex I dysfunction in dopaminergic neurons of the substantia nigra in Parkinson’s disease, triggering abnormal aggregation of α-synuclein. This mechanism is closely associated with impaired repair capacity of the thioredoxin system and reduced activity of methionine sulfoxide reductases [26, 27]. In Alzheimer’s disease, sulfur metabolism abnormalities inhibit β-amyloid clearance pathways and impair mitochondrial electron transport chain function, resulting in ROS accumulation and the hyperphosphorylation of the tau protein [28–30]. In cardiovascular diseases, hyperhomocysteinemia (HHcy) promotes atherosclerosis through three mechanisms: Hcy activates the NF-κB pathway in vascular endothelial cells, inducing the release of inflammatory factors [31]; Hcy mediates thiol modification of low-density lipoprotein (LDL), forming oxidized LDL (ox-LDL) and accelerating foam cell formation; and inhibition of nitric oxide synthase leads to vascular dysfunction, with its pathological basis directly related to defects in the transsulfuration pathway and abnormalities in the folate cycle (vitamin B12-dependent) [32, 33]. In genetic disorders, cystinuria is caused by mutations in SLC7A9/SLC3A1, leading to dysfunction of the cystine/glutamate antiporter (SLC7A11/xCT) and defective renal tubular cystine reabsorption, compounded by insufficient compensatory activity of the NADPH-dependent disulfide reductase system [34, 35]. Methylmalonic acidemia arises from defects in cystathionine β-synthase, causing the accumulation of Hcy and methylmalonic acid (MMA), which inhibit mitochondrial tricarboxylic acid (TCA) cycle activity and succinate dehydrogenase function, exacerbating oxidative stress and energy metabolism collapse [32, 36] (Table 1).

**Table 1:** Key molecules, core pathways and pathophysiological links to diseases in sulfur metabolism.

Category	Metabolic pathway/critical molecule	Physiological function/mechanism	Associated diseases
Sulfur-containing amino acids	Methionine (Met)	Initiator amino acid for protein synthesis; provides methyl donor (SAM) for DNA methylation and polyamine biosynthesis.	Growth retardation, metabolic disorders
	Cysteine (Cys)	Participates in the biosynthesis of iron-sulfur (Fe-S) clusters, hydrogen sulfide, and taurine.	Cystinuria
Core metabolic pathways	Methionine cycle	Generates SAM for methyl group donation; transfers sulfur to Cys via trans-sulfuration to mitigate homocysteine (Hcy) toxicity.	Hyperhomocysteinemia
	Trans-sulfuration pathway	Combines Hcy with serine to form Cys, releasing α-ketobutyrate and ammonia.	Methylmalonic acidemia
	Glutathione (GSH) system	Major antioxidant (GSH/GSSG cycle); scavenges reactive oxygen species (ROS); mediates drug detoxification via glutathione S-transferase (GST)-catalyzed conjugation.	Parkinson’s disease
Redox regulation	Thioredoxin (TRX) system	Repairs oxidized proteins and maintains intracellular redox homeostasis.	Alzheimer’s disease
	Glutathione peroxidase (GPX)	Eliminates H_2_0_2_ and lipid hydroperoxides	Oxidative stress-related disorders
Sulfur metabolism disorders	Homocysteine (Hcy) accumulation	Inhibits vascular endothelial function and promotes thrombosis.	Atherosclerosis
	GSH depletion	Reduces antioxidant capacity, exacerbating oxidative stress.	Parkinson’s disease

### Identification and characterization of disulfidptosis

As early as the last century, THONARTP et al. reported that GSH depletion is due to the inhibition of cystine uptake via the cystine–glutamate reverse transporter system [37]. By 2020, the abnormal accumulation of cystine was shown to induce disulfide stress and a high degree of cytotoxicity, and this mechanism of cytotoxicity differed from that reported previously [38]. In cancer cells with aberrant expression of the cystine transporter SLC7A11, the high rate of cystine uptake and reduction of cystine to Cys, combined with glucose starvation and depletion of the NADPH pool, allows the accumulation of large amounts of intracellular disulfide molecules, which leads to rapid cell death. Disulfidptosis can be distinguished from other modes of death, and the activation of disulfidptosis requires three conditions [39]. The first condition is high expression of SLC7A11 [40]. SLC7A11 transfers cystine into the cell and glutamate to the extracellular space, resulting in high uptake of extracellular cystine, which leads to a large accumulation of intracellular cystine, causing disulfide bond stress in cellular metabolism. The second condition is glucose starvation. When glucose metabolism is blocked, the generation of reduced NADPH via the pentose phosphate pathway is suppressed [41]. The third condition involves the formation of an abnormal disulfide bond between actin backbone proteins. When all three of these conditions are met, the massive accumulation of disulfides accelerates disulfide bonding between actin backbone proteins, causing actin to contract and detach from the plasma membrane, ultimately leading to cell shrinkage and death. Cell shrinkage and death result from the detachment of actin from the plasma membrane [5].

### Mechanisms of disulfidptosis

**Fig. 2:**
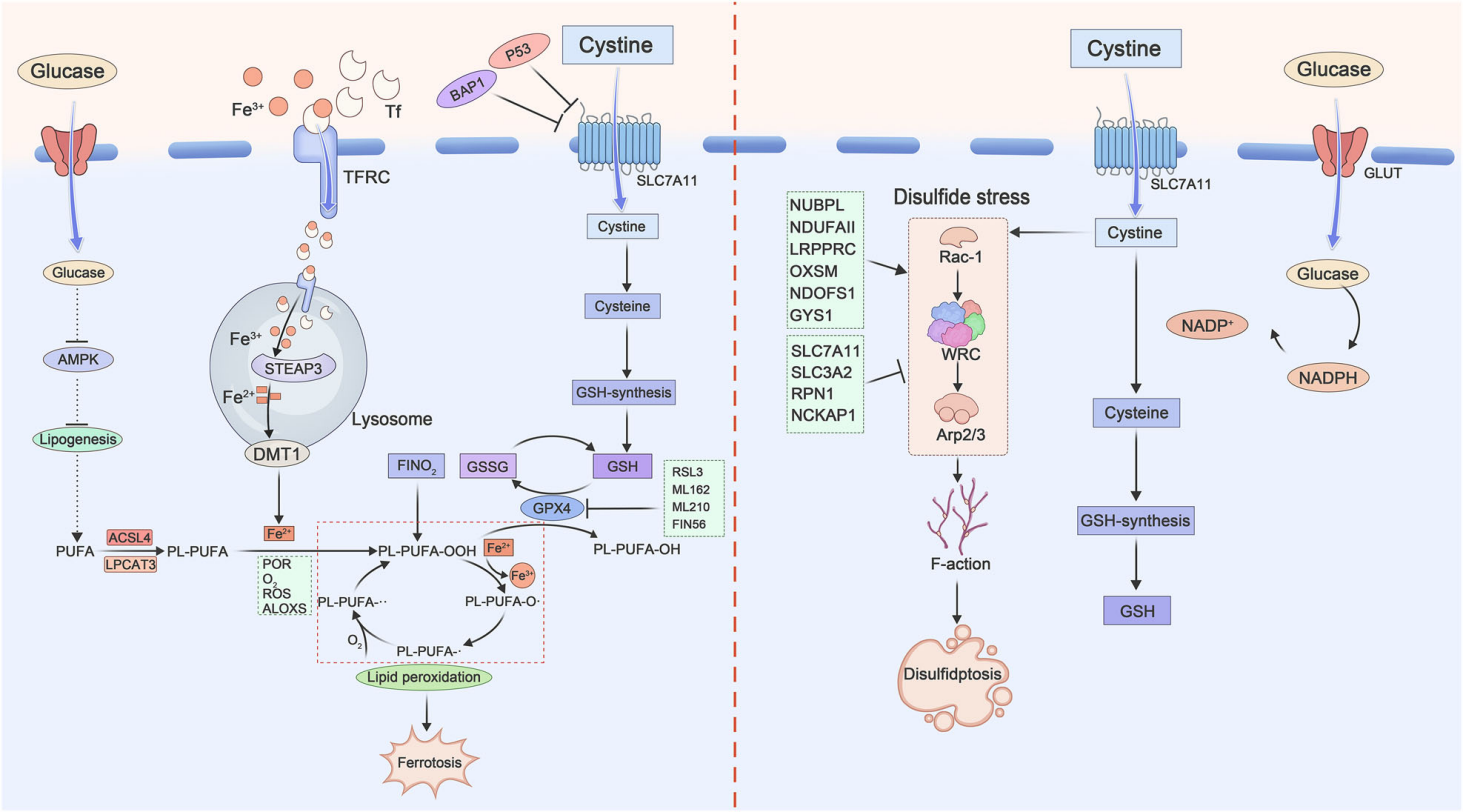
Molecular mechanisms of ferroptosis and disulfidptosis. In the left panel, Fe^3+^ bound to TF and binds to TfR1 on the cell membrane to enter the cell by endocytosis. Excess iron can directly lead to ferroptosis, and ROS is also produced, which promotes the peroxidation of PUFAs on cell membranes. GPX4 can inhibit ferroptosis by reducing lipid peroxidation. As shown in the figure on the right, glucose starvation leads to high uptake of cystine and reduced cysteine, NADPH depletion and disulfide stress due to intracellular accumulation of disulfides, which activates the Rac-WRC-Arp2/3 signaling pathway, leading to disulfide bond abnormalities in actin cytoskeletal proteins and disulfidptosis.

Liu et al. [5] demonstrated that SLC7A11-high tumor cells undergo disulfidptosis under glucose starvation, independent of apoptosis/ferroptosis but reversible by reducing agents (e.g., β-mercaptoethanol). Chemical proteomics revealed a 1.5-fold increase in disulfide bonds within actin cytoskeleton proteins, which was abolished by SLC7A11 knockdown. Genome-wide CRISPR-Cas9 screening identified suppressor genes (SLC7A11, SLC3A2, RPN1, and NCKAP1) and synergistic mitochondrial oxidative phosphorylation genes (NUBPL, NDUFA11, and LRPPRC) [42]. NCKAP1 knockdown attenuated disulfide bond formation and F-actin collapse, highlighting the critical role of Rac1-WRC-Arp2/3 signaling in actin polymerization [43]. Glucose transporters (GLUT) inhibitors (e.g., BAY-876) induce disulfidptosis in vitro and suppress tumor growth in vivo (Fig. 2).

Recent studies have provided mechanistic insights: Yao et al. [44] identified the lncRNA CASC8 as a disulfidptosis inhibitor in pancreatic ductal adenocarcinoma (PDAC). CASC8 stabilizes c-Myc to activate the pentose phosphate pathway, increasing NADPH levels to counteract disulfide stress. Conversely, Wang et al. [45] revealed that ER stress activation via PERK/eIF2α/ATF4 signaling protects against disulfidptosis, whereas ER stress inhibitors (e.g., GSK2656157) synergize with GLUT1 antagonists to exacerbate disulfide accumulation and tumor suppression. Shi et al. [46] demonstrated that gaudichaudione H induces disulfidptosis in hepatocellular carcinoma (HCC) by stabilizing NRF2 to upregulate SLC7A11, depleting GSH and NADPH. Similarly, Tang et al. [47] reported that thioredoxin reductase 1 (TrxR1) inhibition triggers disulfidptosis in glioblastoma (GBM) by disrupting redox homeostasis, leading to cytoskeletal collapse and immunogenic cell death. Collectively, these studies elucidate the core regulatory mechanisms underlying disulfidptosis. The SLC7A11 transporter, functioning as a cystine/glutamate antiporter, facilitates cystine uptake under glucose deprivation conditions, resulting in NADPH depletion and subsequent disulfide accumulation that ultimately induces cell death. Metabolic reprogramming processes, particularly those involving the pentose phosphate pathway, GSH metabolism, and NADPH biosynthesis, constitute critical regulatory nodes determining cellular sensitivity to disulfidptosis. Furthermore, diverse molecular regulators, including long non-coding RNA CASC8, endoplasmic reticulum stress responses, the natural compound gambogenic acid, and TrxR1, modulate disulfidptosis through mechanisms involving transcriptional regulation, redox homeostasis maintenance, and autophagy modulation, collectively underscoring the pathophysiological complexity of this process. Notably, tissue-specific manifestations across pancreatic ductal adenocarcinoma, HCC, and glioblastoma suggest broad therapeutic potential for disulfidptosis modulation. A key scientific advancement lies in the discovery that TrxR1 inhibition elicits NRF2-dependent disulfidptosis accompanied by immunogenic cell death characteristics, providing a novel mechanistic rationale for combination immunotherapy strategies.

### Relationships between disulfidptosis and other types of programmed cell death

**Fig. 3:**
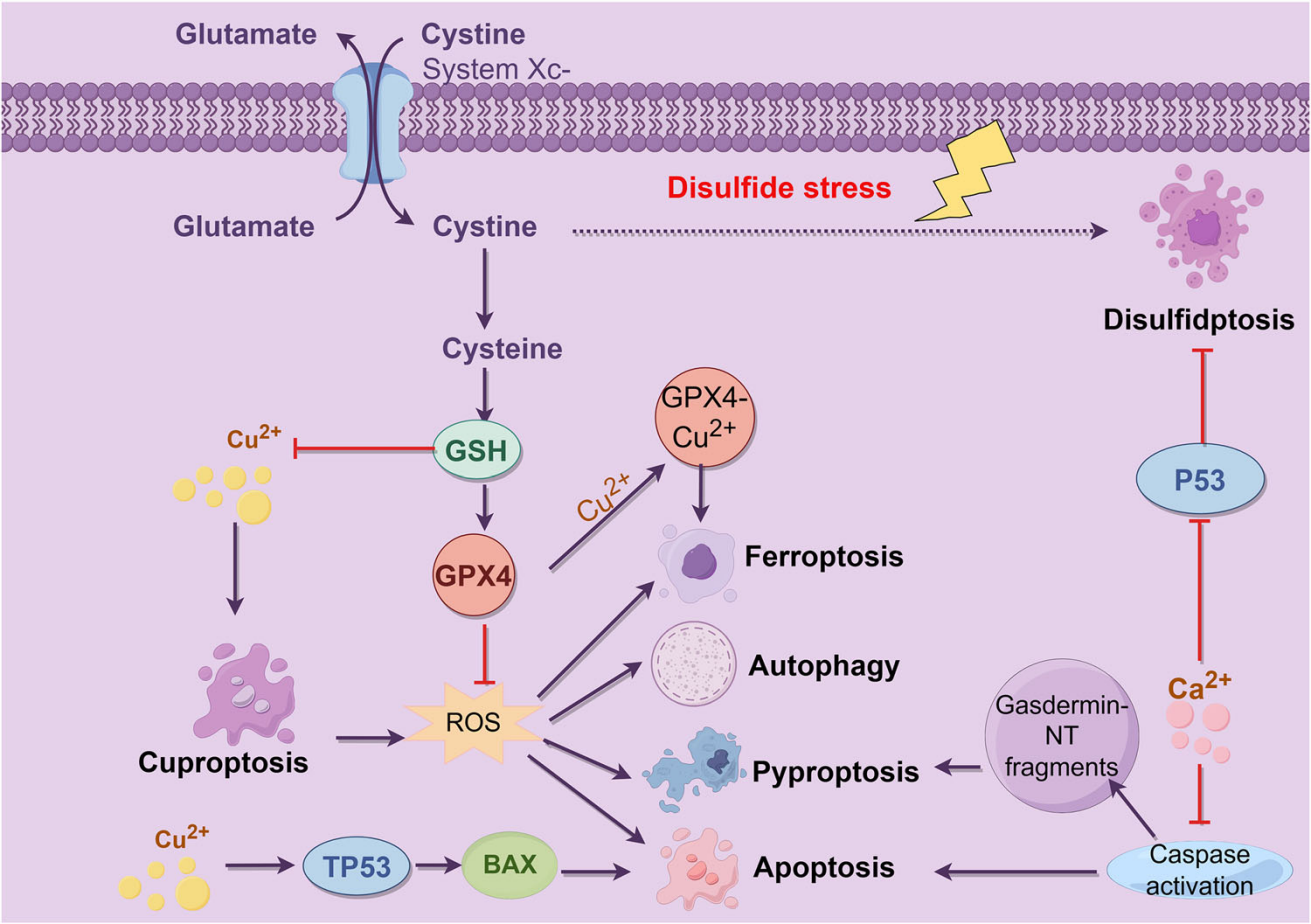
Relationship between disulfidptosis and other types of programmed cell death. This figure was created with Figdraw.

As cellular research advances, emerging evidence reveals intricate crosstalk between disulfidptosis and other RCD modalities, including ferroptosis, cuproptosis, and apoptosis (Fig. 3). Disulfidptosis, which is uniquely driven by SLC7A11-mediated cystine overload and NADPH depletion, intersects with these pathways through shared molecular mechanisms and metabolic dependencies (Table 2).

**Table 2:** Relationships between disulfidptosis and other types of programmed cell death.

Cell death type	Regulatory mechanism	Key molecules	Morphological features	Association with disulfidptosis
Disulfidptosis	Excessive cystine uptake mediated by SLC7A11 depletes NADPH, causing disulfide stress and cytoskeletal collapse	SLC7A11, NADPH, actin	Plasma membrane rupture, no apoptotic bodies	Self-core mechanism
Ferroptosis	SLC7A11 imports cystine to support glutathione synthesis, enabling GPX4 to inhibit lipid peroxidation	SLC7A11, GPX4, GSH	Iron-dependent lipid peroxidation	SLC7A11 competition, GSH depletion synergy
Cuproptosis	Copper ion overload induces mitochondrial protein aggregation and dysfunction	Cu²⁺, GSH	Mitochondrial dysfunction	GSH depletion promotes cuproptosis; copper inhibits GPX4 to exacerbate ferroptosis
Apoptosis	Caspase activation leads to mitochondrial outer membrane permeabilization and chromatin condensation	Caspases, Bcl-2 family	Nuclear condensation, apoptotic bodies	Thiol oxidants trigger both; GSDME cleavage induces membrane rupture
Pyroptosis	Gasdermin proteins form plasma membrane pores and release inflammatory cytokines IL-1β/IL-18	GSDMD, NLRP3	Plasma membrane pores, inflammation	NINJ1 amplifies membrane damage in both
Necroptosis	RIPK3/MLKL kinase signaling mediates plasma membrane permeabilization	RIPK3, MLKL	Kinase-dependent membrane permeabilization	Oxidative stress initiates both, distinct execution
Metabolic vulnerabilities	All pathways involve redox imbalance and energy metabolism regulation	NADPH, mitochondrial function	Energy metabolism dysfunction	Combination therapy enhances anti-tumor effects

#### Disulfidptosis and ferroptosis

SLC7A11 serves as a critical node in both pathways. In ferroptosis, SLC7A11 imports cystine for GSH synthesis, which supports GPX4-mediated antioxidant defense to inhibit lipid peroxidation. Conversely, under glucose deprivation, high SLC7A11 expression drives excessive cystine uptake, leading to NADPH depletion and disulfide stress, thereby inducing disulfidptosis. Both pathways involve redox imbalance but diverge in execution: ferroptosis is iron-dependent and characterized by lipid peroxidation, whereas disulfidptosis arises from disulfide bond accumulation in cytoskeletal proteins. Notably, ferroptosis inducers (e.g., erastin) may sensitize cells to disulfidptosis by depleting GSH [48].

#### Disulfidptosis and cuproptosis

Copper (Cu²⁺) overload, a hallmark of cuproptosis, intersects with disulfidptosis through GSH dynamics [49]. GSH depletion during disulfidptosis elevates free Cu²⁺, which promotes cuproptosis by inducing mitochondrial protein aggregation. Conversely, copper overload inhibits GPX4, exacerbating ferroptosis and indirectly priming cells for disulfidptosis. Copper overload also induces ROS, exacerbating disulfide stress and membrane damage in disulfidptosis.

#### Disulfidptosis and apoptosis

Apoptosis is caspase-dependent and involves mitochondrial outer membrane permeabilization or death receptor signaling, whereas disulfidptosis is caspase-independent and driven by cytoskeletal collapse due to disulfide stress. However, thiol oxidants (e.g., diamide) can trigger both pathways by disrupting redox balance. Caspase-3 cleavage of GSDME during apoptosis may transition from cell death to pyroptosis or disulfidptosis-like membrane rupture [50].

#### Disulfidptosis and pyroptosis

Both involve plasma membrane permeabilization. Pyroptosis relies on gasdermin family proteins forming plasma membrane pores and releasing IL-1β/IL-18 [51]. Disulfidptosis involves actin cytoskeleton disulfide cross-linking and subsequent membrane rupture, independent of gasdermins. NINJ1, a membrane protein, amplifies membrane damage via both pathways [52].

#### Disulfidptosis and necroptosis

Necroptosis is RIPK3/MLKL dependent, while disulfidptosis is regulated by SLC7A11 and NADPH metabolism. Both may be initiated by oxidative stress but diverge in execution: necroptosis involves kinase-mediated membrane permeabilization, whereas disulfidptosis results from disulfide stress in the actin cytoskeleton [53].

#### Shared metabolic vulnerabilities

All these pathways converge on redox and metabolic regulation. Disulfidptosis and ferroptosis both rely on lipid peroxidation, albeit through distinct mechanisms (disulfide stress vs. iron-dependent peroxidation) [54]. Cuproptosis and ferroptosis are associated with mitochondrial dysfunction, whereas apoptosis and pyroptosis are modulated by caspase activation. These interconnections suggest that disulfidptosis inducers combined with ferroptosis modulators (e.g., GPX4 inhibitors) or copper chelators (e.g., tetrathiomolybdate) could exploit metabolic vulnerabilities in tumors.

## Disulfidptosis and cancer

**Fig. 4:**
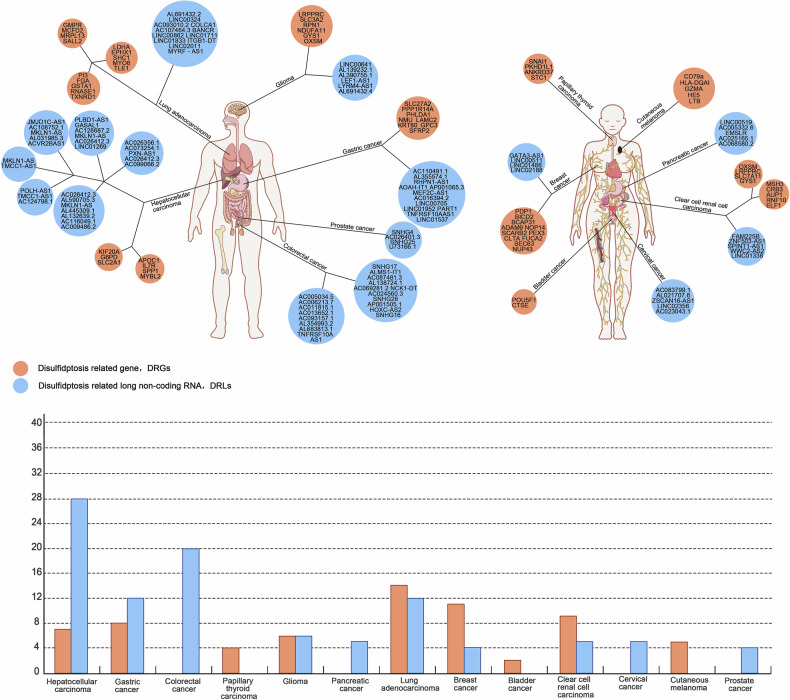
Statistics of genes and non-coding RNAs related to disulfidptosis in various types of tumors. The histogram represents the number of DRGs and DRLs for each tumor.

Malignant tumors pose a great threat to human health and remain an important global healthcare issue. Disulfidptosis is a new discovery that reveals a new mechanism by which organisms fight malignant tumors. It is difficult to detect actin contraction and detachment from the plasma membrane under physiopathological conditions in humans. Studying the relationships of these disulfidptosis-related genes with different tumors is necessary. Long noncoding RNAs (lncRNAs) are a class of RNA molecules more than 200 nucleotides in length that regulate gene expression at the pretranscriptional, transcriptional, and posttranscriptional levels [55]. An increasing number of studies have shown a strong association between lncRNAs and various forms of cancer, and these lncRNAs can be used as important predictors of tumor prognosis [56]. Disulfidptosis-related long noncoding RNAs (DRLs) have potential prognostic value (Fig. 4).

### Pancancer research

In most tumor types, the distribution of disulfidptosis levels is different. The classification of high/low Disulfidptosis score is the result of an integrated multidimensional analysis covering gene expression, metabolic status, molecular variants, clinical prognosis and functional experimental validation (Table 3). Low-grade gliomas, glioblastomas, lymphoid tumors diffuse large B-cell lymphoma and acute myeloid leukemia are associated with lower rates of death by disulfidocytosis [57]. Different tumors have different sensitivities to disulfidptosis, and these differences can be analyzed to understand the types of tumors that are sensitive to disulfidptosis, for which disulfidptosis treatment has better results. Moreover, different tumor types were analyzed, and it was found that tumor types of the nervous system have higher levels of disulfidptosis than do tumor types of the blood system. Melanoma cells are cell types with high disulfidptosis scores, and in related experiments, the use of the disulfidoptosis bond reducer dithiothreitol (DTT) eliminated cell death caused by inducing glucose starvation [57]. An investigation of the differences in DRG expression revealed that SLC7A11 had the lowest average expression, GYS1 and LRPPRC had moderate expression, and RPN1 and SLC3A2 had high expression [42, 43]. The most significant survival biomarker identified in the analysis of differences in DRG expression was SLC7A11. A significant difference in the survival status of eight tumors was observed between the low-expression SLC7A11 group and the high-expression SLC7A11 group on the basis of K‒M analysis of the low- versus high-DRG groups [43]. This difference was greater than the variances observed in the other DRG groups. These results collectively suggest that SLC7A11 may be the most prognostically relevant DRG for predicting survival status across various cancers. Among the 32 human malignancies containing disulfidptosis genes, LRPPRC emerged as the most frequently mutated gene, particularly in somatic endometrial carcinoma and cutaneous melanoma, with mutation rates exceeding 15%. Additionally, a significant correlation was noted between the mesenchymal scores of low-grade gliomas in the brain and the expression of GYS1 and RPN1. Further correlation analysis of the tumor microenvironment revealed a negative correlation between LRPPRC and immunity across all tumors. In a pancancer study, LRPPRC and SLC7A11 were found to be positively correlated with TMB in most gastrointestinal tumors, suggesting their significant roles in gastrointestinal cancers [42].

**Table 3:** Analysis of disulfidptosis score in cancer types: molecular features and clinical correlations.

Tumor type	Disulfidptosis score	Key evidence
Low-grade glioma (LGG)	High-score	- High SLC7A11 expression, low GLUT1 expression - Reduced glucose metabolic flux - Longer survival
Glioblastoma (GBM)	High-score	- RAC1 amplification - Sensitivity to glucose deprivation - Increased CD8+ T-cell infiltration
Prostate adenocarcinoma (PRAD)	High-score	- PTEN deletion - Abnormal methylation regulation
Skin cutaneous melanoma (SKCM)	High-score	- High SLC7A11 expression correlates with favorable prognosis - Sensitive to Etoposide - Frequent FLNA/FLNB mutations
Pancreatic adenocarcinoma (PAAD)	High-score	- High DS Score significantly associated with prolonged OS - EMT pathway inhibition
Acute myeloid leukemia (LAML)	Low-score	- Low SLC7A11 expression, high GLUT1 expression - Dependency on oxidative phosphorylation - Reduced sensitivity to Cytarabine
Diffuse large B-cell lymphoma (DLBC)	Low-score	- Active glucose metabolism - Immunosuppressive microenvironment
Ovarian cancer (OV)	Low-score	- SLC7A11 mutations linked to poor prognosis - CDKN2A deletion - Resistance to PARP inhibitors
Stomach adenocarcinoma (STAD)	Low-score	- Low LRPPRC expression predicts lymph node metastasis - Increased Treg infiltration - Resistance to GW-441756
Head and neck squamous cell carcinoma (HNSC)	Low-score	- Methylation-driven PDLIM1 dysregulation - Poor response to immune checkpoints - High stemness features
Colon adenocarcinoma (COAD)	Mixed (subtype-dependent)	- MSI-H subtypes may be high-score - Majority are low-score
Breast invasive carcinoma (BRCA)	Heterogeneous	- Luminal subtypes: High-score - Basal subtypes: Low-score - Significant drug sensitivity variability across subtypes

Glucose depletion-induced disulfidptosis in cells is associated with increased drug sensitivity, indicating potential implications for disulfidptosis-related therapy. Experimental studies demonstrated a positive correlation between NCKAP1 and SLC7A11 and drug sensitivity to targeted drugs, whereas GYS1 showed a negative correlation [42]. NCKAP1 was identified as a crucial DRG, with previous studies highlighting its key roles in immunotherapy and chemotherapy [58, 59]. Moreover, NCKAP1, a gene that regulates the actin cytoskeleton, was found to influence tumors by forming regulatory complexes (Table 4).

**Table 4:** The different features of high-score tumors and low-score tumors.

Feature	High-score tumors	Low-score tumors
Representative types	Low-grade glioma (LGG), Glioblastoma (GBM), Prostate adenocarcinoma (PRAD), Melanoma (SKCM)	Acute myeloid leukemia (LAML), Diffuse large B-cell lymphoma (DLBC), Ovarian cancer (OV), Stomach adenocarcinoma (STAD)
Molecular profile	- High SLC7A11 expression - Low GLUT1 expression - Reduced glucose metabolic flux	- Low SLC7A11 expression - High GLUT1 expression - Oxidative phosphorylation dependency
Prognosis	Longer survival	Poor survival
Drug sensitivity	Sensitive to Cytarabine (AML), Etoposide (SKCM), Methotrexate (BRCA)	Resistant to GW-441756 (RTK inhibitor), ABT-263 (BCL-2 inhibitor)
Immune microenvironment	Increased CD8+ T cells, B cells Decreased Tregs, M0 macrophages	Increased Tregs, M0 macrophages Enhanced response to CTLA4 inhibitors
Mutation/CNV features	Frequent FLNA/FLNB mutations (UCEC/SKCM) SLC3A2 amplification (BRCA/LUSC)	PTEN deletion (PRAD) CDKN2A deletion (KIRC/LGG)

### Prognostic modeling and clinical validation of the prognostic models

In prognostic modeling, different predictive models use different techniques. Most predictive models are built by analyzing genes via techniques such as Pearson analysis, differential analysis, and Cox regression. The most important test for predictive models is the area under the ROC curve (AUC), which is helpful for predicting the prognosis of patients. NCKAP1, BRK1, ACTR2, ACTR3, and RAC1 [60] had higher AUCs than most other predictive models did, suggesting a better role. In addition, the results of the nomogram of this model also indicate its better predictive ability. Another model related to DRLs in breast cancer also has better predictive ability [61].

After a predictive model with good predictive ability is built, a risk assessment system can be developed to classify tumor patients into high-risk and low-risk groups on the basis of the characteristic genes in the model. A study of the risk assessment system revealed significant differences in clinical outcomes between patients in the high-risk group and those in other groups, as evidenced by the analysis of the TCGA and test datasets. A correlation between the overall survival time of high-risk patients identified by the risk assessment system and the DRG score was observed, indicating that higher DRG scores were associated with increased risk and decreased overall survival time.

Prognostic models constructed using DRGs and DRLs have been used in HCC [62–69], gastric cancer [70–72], colorectal cancer [73, 74], papillary thyroid cancer [75], glioma [76, 77], pancreatic cancer [78], lung adenocarcinoma [79–82], breast cancer [83, 84], bladder cancer [85], renal clear cell carcinoma [86–88], cervical carcinoma [89], cutaneous melanoma [90] and prostate cancer [91], and the constructed prognostic models are reliable and accurate. The establishment of predictive models helps to reveal the molecular mechanism and clinical significance of disulfidptosis in patients with tumors and provides a basis for developing more precise and effective treatment strategies (Table 5).

**Table 5:** Disulfidptosis-related genes and non-coding RNAs in various types of tumors.

Tumor	Disulfidptosis related gene	Disulfidptosis related non-coding RNA
Hepatocellular carcinoma	KIF20A, G6PD, SLC2A1, APOC1, IL7R, SPP1, MYBL2	MKLN1-AS, AC026412.3, LINC01269, JMJD1C-AS1, AC108752.1, AL031985.3, ACVR2BAS1, MKLN1-AS, TMCC1-AS1, POLH-AS1, TMCC1-AS1, AC124798.1, AC026412.3, AL590705.3, AL445309.1, AL132639.2, AC116049.1, AC009486.2, AC026356.1, AC073254.1, PXN-AS1, AC026412.3, AC099066.2
Gastric cancer	SLC27A2, PPP1R14A, PHLDA1, LAMC2, KRT80, NMU, GPC3, SFRP2	AC110491.1, AL355574.1, RHPN1-AS1, AOAH-IT1, AP001065.3, MEF2C-AS1, AC016394.2, LINC00705, LINC01952, PART1, TNFRSF10AAS1, LINC01537
Colorectal cancer		SNHG17, ALMS1-IT1, AC087481.3, AL138724.1, AC069281.2, NCK1-DT, AC024560.3, SNHG26, AP001505.1, HOXC-AS2, SNHG16, AC005034.5, AC006213.7, AC011815.1, AC013652.1, AC093157.1, AL354993.2, AL683813.1, TNFRSF10A-AS1
Papillary thyroid carcinoma	SNAI1, STC1, PKHD1L1, ANKRD37	
Glioma	LRPPRC, SLC3A2, RPN1, NDUFA11, GYS1, OXSM	LINC00641, AL139232.1, AL390755.1, LEF1-AS1, LYRM4-AS1, AL691432.4,
Pancreatic cancer		LINC0051, AC005332.6, EMSLR, AC025165.1, AC068580.2
Lung adenocarcinoma	EPHX1, LDHA, SHC1, MYO6, TLE1, GMPR, MCFD2, MRPL13, SALL2, FGA, GSTA1, PI3, RNASE1, TXNRD1	AL691432.2, LINC00324, AC093010.2, COLCA1, AC107464.3, BANCR, LINC00862, LINC01711, LINC01833, ITGB1-DT, LINC02011, MYRF-AS1
Breast cancer	PDP1, BICD2, BCAP31, ADAM9, NOP14, SCARB2, PEX3, CLTA, FUCA2, SEC63, NUP43	GATA3-AS1, LINC00511, LINC01488, LINC02188
Bladder cancer	POU5F1, CTSE	
Clear cell renal cell carcinoma	LRPPRC, SLC7A11, OXSM, GYS1, MSH3, CRB3, AUP1, RNF10, ELF1	FAM225B, ZNF503-AS1, SPINT1-AS1, WWC2-AS2, LINC01338
Cervical cancer		ZSCAN16-AS1, AC083799.1, AL021707.6, LINC02356, AC023043.1
Cutaneous melanoma	cD79a, HLA-DQAI, GZMA, HE5, LTB,	
Prostate cancer		AC026401.3, SNHG4, SNHG25, U73166.1

### Hepatocellular carcinoma

In the prediction model established by Chen et al. [69], CD8A was the gene with the strongest correlation with tumor immune cells among the 6 genes, suggesting that CD8A plays a critical role in disulfidptosis and immunity and that CD8A may be a bridge linking disulfidptosis and immunity. The study of its potential interactions could elucidate the relationship between disulfidptosis and immunity, leading to the development of new therapeutic modalities. SLCO1B1 is a key gene in the Wang et al. prediction model [92]. The role of SLCO1B1 in cells, which encodes a transporter protein located on the cell membrane, is decreased in HCC and is involved in the entry of chemotherapeutic agents into the cell [93]. Furthermore, SLCO1B1 overexpression prevented HCC cells from proliferating, migrating, and invading. The predictive model established with immune checkpoint genes is considered strongly correlated with disulfidytic acid-mediated apoptosis and may play a significant role in enhancing tumor immunity, making it a useful predictor of HCC prognosis.

A study of MKLN1-AS revealed that it could regulate HCC cells through multiple pathways [64, 65, 68]. First, MKLN1-AS can increase the expression of hepatocellular carcinoma-derived growth factors, which compete with miR-654-3p for endogenous RNAs and promote HCC progression through the MKLN1-AS/hepatic-derived growth factor pathway [94]. Guo et al. demonstrated that MKLN1-AS can also act as an upstream factor of yes-related transcriptional regulator 1, further contributing to HCC progression [95]. Additionally, the SOX9/MKLN1-AS axis has been identified as closely related to tumor proliferation in HCC cells. These experimental results collectively suggest that MKLN1-AS plays a crucial role in the malignant progression of tumors [96]. Furthermore, the integration of MKLN1-AS into predictive models can effectively identify relevant patients, thereby aiding in patient survival and prognosis. These findings highlight the potential of MKLN1-AS as a future therapeutic target in the management of HCC.

### Gastric cancer

NCKAP1 is a core gene in the model established by Yan et al. [72]. Experimental studies are needed to correlate the role of NCKAP1 in gastric cancer, which may affect the progression of gastric cancer through disulfidptosis. NCKAP1 was found to inhibit the malignant behavior of renal clear cell carcinoma cells in studies of renal clear cell carcinoma, suggesting its potential as a prognostic biomarker for screening patients in clinical practice. Deletion of NCKAP1 can alter the spreading and adhesion patch dynamics of the primary actin nucleating agent involved in plate pseudopod formation in fibroblasts.

### Colorectal cancer

Xiao et al. [97] reported that among the characterized genes associated with disulfidptosis, KIF7 plays a crucial role in predictive modeling. Studies have shown that low KIF7 expression is associated with poor prognosis in epithelial ovarian cancer, and KIF7 has also been reported to inhibit basal cell carcinoma [98, 99]. Another significant gene, GDI1, is involved in regulating the GDP/GTP exchange response of the Rab family within cells. High expression of GDI1 in colorectal cancer patients has been linked to a poor prognosis, and research on colorectal cancer cells has demonstrated its ability to promote cell proliferation and migration [100]. Therefore, GDI1 in colorectal cancer serves as an important prognostic biomarker that can guide clinical prognosis and treatment.

ZEB1-AS1 is closely associated with colon cancer, with studies indicating its high expression in colon cancer and its correlation with poor prognosis [101]. The promotion of colon cancer progression by ZEB1-AS1 is attributed to its aberrant expression in cells, as it is typically low in normal tissues [102]. Mechanistic studies revealed that ZEB1-AS1 regulates tumors through the miR-101/ZEB1 axis. A comparison of ZEB1-AS1 expression with clinical stage suggested that ZEB1-AS1 plays a role in promoting tumor progression [103]. Experimental and clinical evidence supports the use of ZEB1-AS1 as a biomarker, enhancing predictive modeling and guiding clinical treatment strategies.

### Papillary thyroid cancer

The predictive models constructed by Liao et al. [75] included SNAI1, STC1, PKHD 1L1 and ANKRD 37, and the differences in the expression of SNAI1 and STC1 were statistically significant across patients. Two important genes in the prediction model are SNAI1 and STC1. SNAI1 has been reported in pancreatic, lung, and colon cancers and is involved in regulating the EMT process in pancreatic tumor cells; its high expression in lung cancer causes distant metastasis in lung cancer; and it has a role in inducing tumor stemness in colon cancer. SNAI1 is also closely related to the stemness and migration of tumor cells, and the overexpression of SNAI1 can significantly increase the malignant biological manifestations of tumors. Another gene, STC1, plays a role in promoting tumor metastasis in ovarian cancer, and the lipid metabolism and cisplatin resistance of tumor cells are also associated with STC1 [104]. In breast cancer, STC is involved in breast cancer proliferation and has been reported to be involved in tumor immune resistance [105]. SNAI1 and STC1 in papillary thyroid cancer need to be further investigated to determine whether they can be used as predictors to guide the prognosis as well as the treatment of papillary thyroid cancer.

### Glioma

In the predictive model constructed by Wang et al. [76], RPN1 and GYS1 expression was elevated and inversely connected with a favorable prognosis, whereas the expression of LRPPRC1 was downregulated in gliomas and positively correlated with a good prognosis. GYS1 is one of the major regulators of glycogen synthesis, and the inhibition of GYS1 leads to the accumulation of glycogen in glioblastoma cells, resulting in the inhibition of proliferation and migration and the formation of ROS, which suggests that the inhibition of GYS1 may be a promising therapeutic target for gliomas [106]. NFE2L3 is not only involved in cell cycle regulation but also associated with apoptosis, proliferation, and inflammatory responses in tumor cells, suggesting that NFE2L3 might be involved in the apoptosis, proliferation and inflammatory responses of tumor cells. NFE2L3 is not only involved in cell cycle regulation but also associated with the apoptosis, proliferation and inflammation of tumor cells, suggesting that it may be involved in disulfidptosis-related processes [107].

In addition, Chen et al. [77] demonstrated that LINC00641 has a unique functional profile and clinical significance in a variety of cancers, including cervical [108] and colorectal cancers [109]. Low-level expression of LINC00641 was observed in glioma cell lines. It interferes with glioma growth through the miR-4262/NRGN pathway [110]. Wang et al. demonstrated that the LYRM4-AS1 protein was able to affect IL-1β-induced chondrocyte growth, suggesting that LEF1-AS1 expression is associated with a poor prognosis in glioma patients [111].

### Pancreatic cancer

DSG3 is an important gene in the predictive model established by Wu et al. [112]. In a related study of DSG3, DSG3 was closely associated with the level of immune cell infiltration, which may suggest a link between DSG3 in disulfidptosis and immunity. An analysis of DSG3 in patients with pancreatic cancer (PC) revealed that it is correlated with clinical stage and that the higher the expression of DSG3 is, the worse the prognosis of PC patients. In PC in vitro cellular assays, DSG3 expression was found to be elevated in tumor tissues compared with normal tissues. The results of the cell migration and scratch assays revealed that DSG3 could enhance the invasive and migratory ability of PC cells [112].

Among the five lncRNAs used to construct predictive models [78], Xing et al. constructed a pyroptosis-associated signature that included LINC00519 and AC005332.6. In addition, LINC00519 was upregulated in tumor tissues and promoted cancer progression.

### Lung adenocarcinoma

According to the prediction model of lung cancer, certain genes are distinctly associated with this disease. TXNRD1, identified in a separate lung cancer prediction model, was found to be correlated with resistance to ferroptosis in tumor cells [81]. In vitro experiments have shown that TXNRD1 influences various malignant biological behaviors in LUAD. KRT18, a cytoskeletal protein, plays a role in non-small cell lung cancer, as knocking down KRT18 results in decreased migratory ability of tumor cells.

In the predictive modeling of DRLs associated with lung cancer, several DRLs have been linked to the disease. Studies have indicated that the lncRNA LINC01833 is associated with poor tumor prognosis [82]. Further exploration revealed that LINC01833 promotes lung cancer metastasis by interacting with miR-519 e-3p. Another significant lncRNA in prediction models is ITGB1-DT, which has been extensively studied in relation to lung cancer [113]. In vitro experiments revealed that the overexpression of ITGB1-DT led to increased malignant behaviors in lung cancer cells, whereas the knockdown of ITGB1-DT reduced these behaviors. Additionally, patient survival has been found to be correlated with ITGB1-DT expression in clinical settings. The discussion surrounding these two DRLs in lung cancer suggests that the predictive model effectively reflects real-world scenarios and can guide patient prognosis and treatment decisions [114].

### Breast cancer

NOP14 is an important gene in the biochemical analysis of breast cancer [84]. In a related study, NOP14 was experimentally examined in PDAC cells, revealing that NOP14 functions as an oncogene that enhances the malignant behavior of PDAC cells. Mechanistic studies have shown that NOP14 is a functional target of mutp53, contributing to tumor invasion and metastasis by increasing the stability of mutp53 mRNA in cells [115].

In the context of breast cancer, distinguishing its subtypes can have significant clinical implications for patient treatment. In a model established by Xia et al., different DRLs were found to be differentially expressed across various breast cancer subtypes. Specifically, LINC00511 and LINC01488 presented relatively high expression levels in the basal subtype [116], whereas GATA3-AS1 was most highly expressed in the normal subtype and Her2. LINC02188 and GATA3-AS1 were significantly more highly expressed in the LumA and LumB subtypes than in the other DRLs.

### Bladder cancer

POU5F1 and CTSE are important genes in the model established by Chen et al. [85]. qRT‒PCR, immunoblotting and immunohistochemical analyses revealed increased expression of CTSE in BCa tumor tissues. Several phenotypic tests have demonstrated the carcinogenic potential of CTSE in BCa cells. POU5F1 transactivates CTSE and promotes BCa cell proliferation and metastasis. In the prediction model established with four genes, SLC7A11, NCKAP 1, SLC3A2, and RPN1 [117], in bladder cancer, high SLC7A11 expression was closely associated with cisplatin resistance, and knockdown of NCKAP1, which encodes a component of Nck-associated protein 1 and the WAVE regulatory complex (WAVE), inhibited rectal cancer cell migration [118].

### Renal clear cell carcinoma

In a model developed by Peng et al., the identification of ISG20 as an important gene was noteworthy [87]. Research on ISG20 revealed a positive correlation between ISG20 levels and SLC7A11 levels, suggesting that ISG20 is potentially involved in the process of disulfidptosis. The pivotal roles of MSH3 and ISG20 in renal carcinoma warrant further investigation.

SPINT1-AS1 is an important DRL in several predictive models [88]. SPINT1-AS 1 has been linked to advancements in several malignancies, including breast cancer [119] and cervical [120], colorectal [121], and esophageal squamous cell carcinomas, and SPINT 1-AS 1 can be used as a therapeutic target for cancer treatment.

### Cutaneous melanoma

In the disulfidptosis-related prediction model constructed by Zhao et al. [90], CD79A was identified as an important gene through the analysis of gene expression and the integration of clinical data. Patients with oral squamous carcinoma exhibiting high CD79A expression levels have an improved prognosis, indicating the role of CD79A as a protective gene [122]. HLA-DQA1, a key determinant in the prediction model, has been extensively studied in various contexts, with associations with multiple tumor types underscoring its clinical significance [123]. Elevated levels of HLA-DQA1 have been linked to favorable outcomes across different cancers, including breast cancer, where it has strong predictive ability. The combined analysis of CD79A and HLA-DQA1 offers a potential biomarker for cutaneous melanoma, presenting opportunities for prognostic and therapeutic guidance in clinical settings.

### Prostate cancer

AC026401.3 was among the important DRLs in the prediction model constructed by Mulati et al. [91]. Knockdown of AC026401.3 in prostate cancer-associated cell lines decreased tumor cell migration and invasive ability. In plate cloning experiments, AC026401.3 inhibited the formation of clonal colonies. These findings suggest that AC026401.3 is a protective gene, and further mechanistic studies on the effect of AC026401.3 on PCa revealed that, combined with the results of IHC staining of SLC7A11, the decrease in the invasive ability of cells caused by knockdown of AC026401.3 can be associated with disulfidptosis. These findings suggest that AC026401.3 is a DRL with a strong correlation with disulfidptosis, and further studies on AC026401.3 may reveal the specific mechanism by which it affects disulfidptosis.

## Disulfidptosis, the tumor microenvironment and treatment

### Tumor microenvironment

**Fig. 5:**
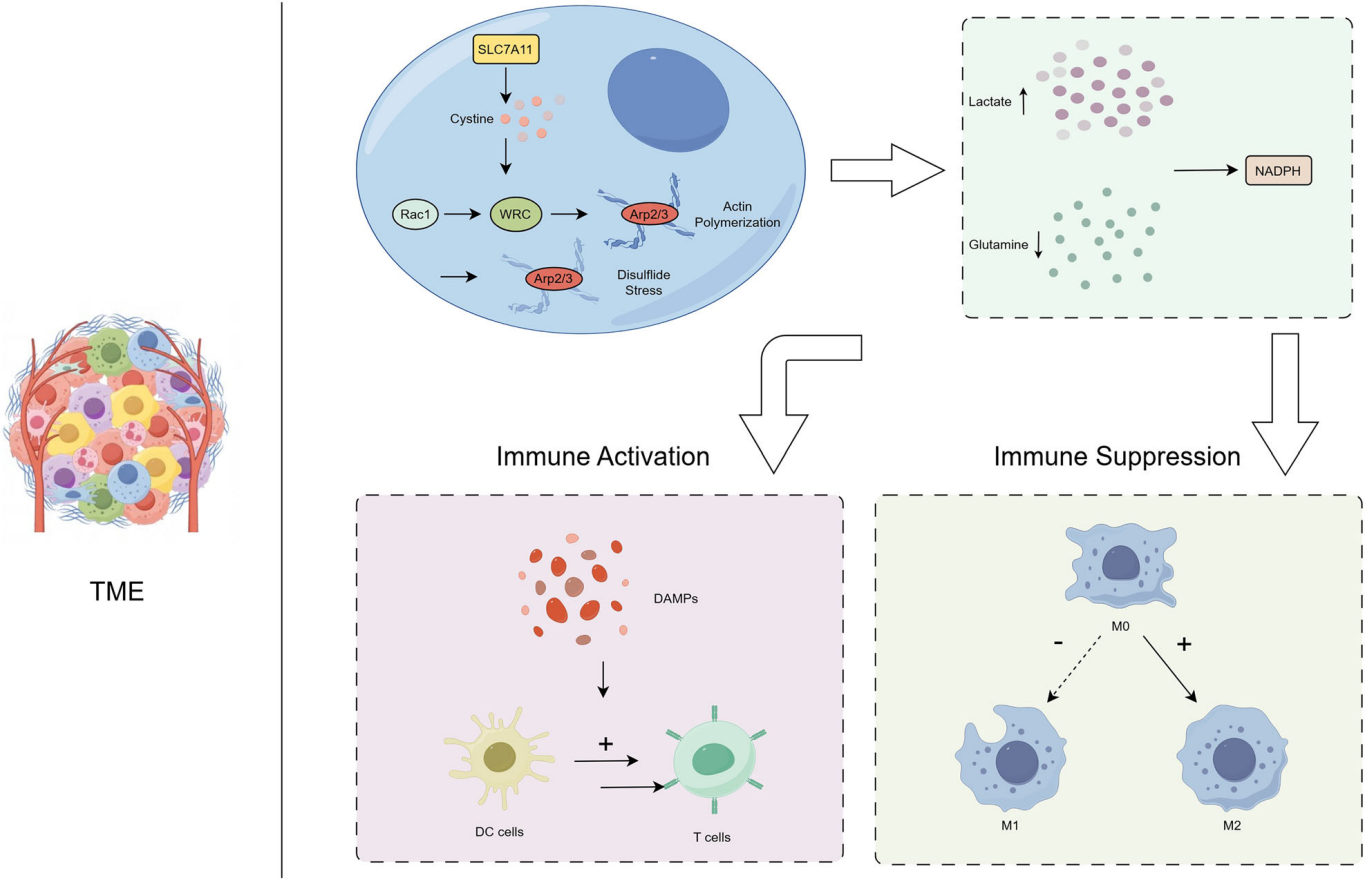
Metabolic-immune axis in tumor microenvironment regulates disulfidptosis. This figure was created with Figdraw.

The tumor microenvironment (TME), composed of tumor cells, stromal cells (e.g., fibroblasts), immune cells (T cells, B cells, macrophages), and extracellular matrix, forms a complex ecosystem where metabolic and immune interactions critically influence tumor progression. Studies reveal that disulfidptosis activity in TME is closely associated with microenvironmental features: subpopulations with high disulfidptosis exhibit reduced mesenchymal infiltration, higher tumor purity, and immunosuppressive characteristics, marked by decreased B cells and CD8⁺ T cells alongside enriched tumor-associated macrophages (TAMs) and regulatory T cells (Tregs) [124]. Metabolically, under glucose deprivation or NADPH depletion, SLC7A11-overexpressing tumor cells undergo disulfidptosis due to cystine accumulation, driven by cytoskeletal collapse via the Rac-WRC-Arp2/3 pathway [5]. Microenvironmental stressors such as lactate accumulation and glutamine depletion exacerbate this process by disrupting NADPH homeostasis [125] (Fig. 5).

Disulfidptosis triggers innate immunity through damage-associated molecular pattern release and dendritic cell (DC) activation, yet excessive activity may promote immunosuppression via Treg recruitment and M2 macrophage polarization [124]. Heterogeneity analyses show that high-risk TMEs display immunosuppressive phenotypes, characterized by TAMs and Tregs secreting inhibitory cytokines (IL-10, TGF-β) and upregulating PD-L1 to suppress CD8⁺ T cell function. In contrast, low-risk TMEs retain intact antitumor immunity with enriched DCs, CD8⁺ T cells, NK cells, and M1 macrophages [125]. Metabolically, high-risk tumors rely on the glucose-pentose phosphate pathway for NADPH production, while low-risk tumors utilize alternative pathways such as serine-folate metabolism [126].

Dysregulated disulfidptosis-related genes (e.g., SLC7A11, RPN1) correlate with poor patient outcomes, whereas low-risk patients show better responses to immune checkpoint inhibitors (e.g., PD-1/PD-L1 inhibitors). Therapeutic strategies targeting SLC7A11 or GLUT selectively induce disulfidptosis in high-risk tumors, and combination with immune checkpoint blockade enhances efficacy by remodeling immunosuppressive TME. Additionally, impaired thioredoxin system function may aggravate disulfide stress, suggesting potential synergy with antioxidant pathway inhibitors [126]. These findings underscore the pivotal role of the TME metabolic-immune axis in disulfidptosis regulation, providing a rationale for stratified combination therapies to improve cancer treatment outcomes (Table 6).

**Table 6:** Metabolic-immune regulatory mechanisms of disulfidptosis in the tumor microenvironment and therapeutic strategies.

Category	Content
TME components	Tumor cells, stromal cells (fibroblasts), immune cells (T cells, B cells, macrophages), extracellular matrix.
Disulfidptosis features	- High-disulfidptosis subpopulations: Reduced mesenchymal infiltration, high tumor purity, immunosuppressive phenotype (decreased B cells and CD8⁺ T cells, enriched TAMs and Tregs). - Low-disulfidptosis subpopulations: Intact antitumor immunity (enriched DCs, CD8⁺ T cells, NK cells, M1 macrophages).
Metabolic mechanism	- Under glucose deprivation or NADPH depletion, SLC7A11-overexpressing tumor cells undergo disulfidptosis due to cystine accumulation, driven by cytoskeletal collapse via Rac-WRC-Arp2/3 pathway. - Microenvironmental stressors (lactate accumulation, glutamine depletion) exacerbate disulfidptosis by disrupting NADPH homeostasis.
Immune response	- Triggers innate immunity via DAMP release and DC activation. - Excessive disulfidptosis promotes immunosuppression through Treg recruitment, M2 macrophage polarization, and secretion of inhibitory cytokines (IL-10, TGF-β) with PD-L1 upregulation.
Heterogeneity analysis	- High-risk TME: Immunosuppressive phenotype (TAMs, Tregs, inhibitory cytokines). - Low-risk TME: Intact antitumor immunity (DCs, CD8⁺ T cells, NK cells, M1 macrophages).
Metabolic pathway differences	- High-risk tumors: Rely on glucose-pentose phosphate pathway (PPP) for NADPH production. - Low-risk tumors: Utilize alternative pathways.
Genes and prognosis	- Dysregulated genes correlate with poor outcomes. - Low-risk patients show better responses to immune checkpoint inhibitors.
Therapeutic strategies	- Target SLC7A11 or GLUT to selectively induce disulfidptosis in high-risk tumors. - Combine with immune checkpoint blockade to remodel immunosuppressive TME. - Synergy with antioxidant pathway inhibitors.
Conclusion	The TME metabolic-immune axis plays a pivotal role in disulfidptosis regulation, providing rationale for stratified combination therapies to improve cancer treatment outcomes.

### Treatment of tumors

Traditional treatments for malignant tumors include radiotherapy, chemotherapy, and surgery. For patients with advanced tumors who are not suitable for surgery, immunotherapy has attracted much attention in recent years. The recent discovery of disulfidptosis is expected to provide a new method for the metabolic treatment of cancer. Currently, there are two main aspects of SLC7A11 that play a therapeutic role in tumors. First, direct inhibition of SLC7A11 transporter protein activity, due to low expression of SLC7A11, promotes ferroptosis to treat tumors. Second, SLC7A11 overexpression can cause intracellular glucose depletion, thereby creating metabolic vulnerability to inhibit tumor progression. In highly expressed SLC7A11 cancer cells, the metabolic vulnerability of tumor cells is induced by a high dependence on glucose and glutamine [92].

**Fig. 6:**
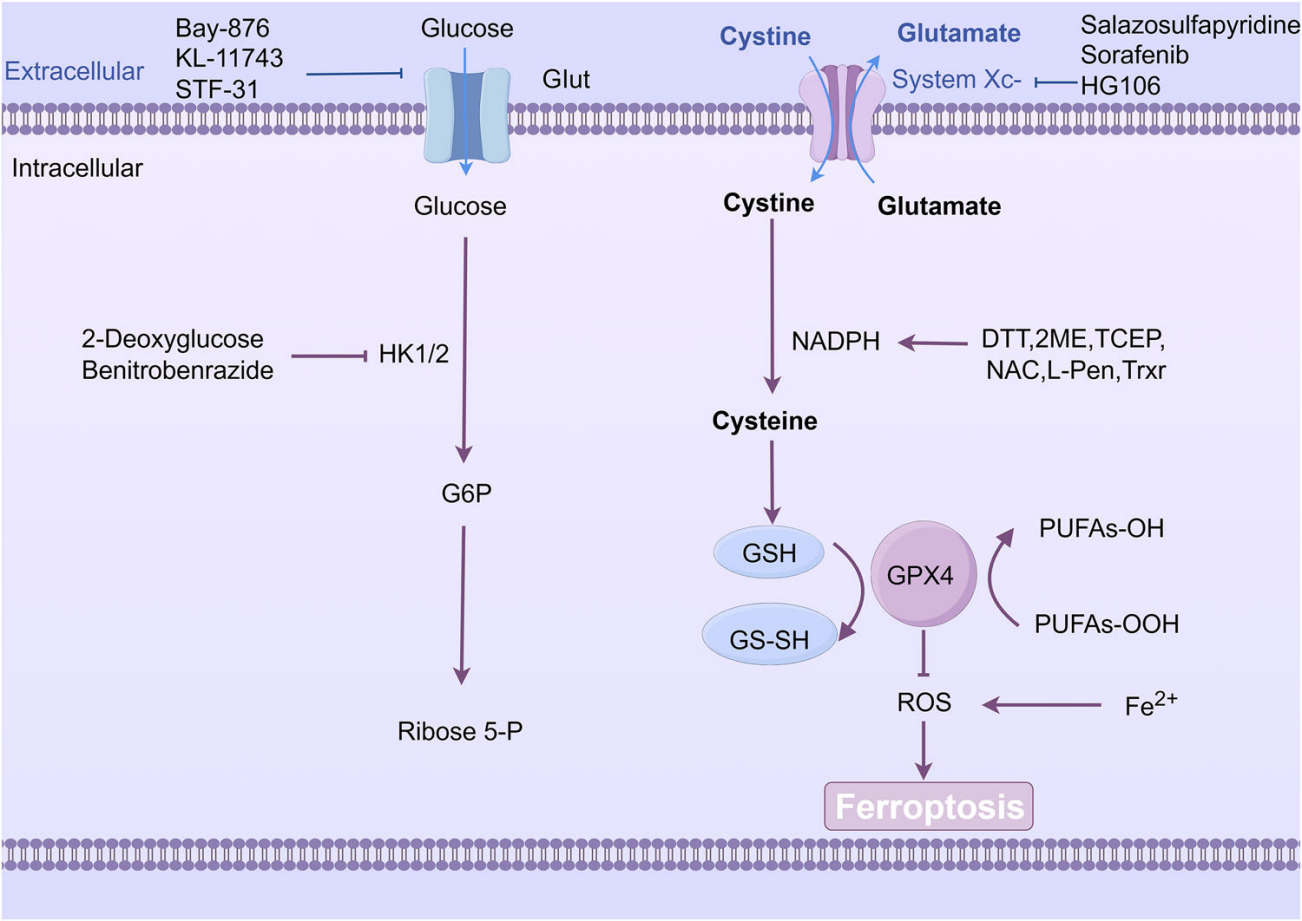
The role of inhibitors and activators in the regulation of disulfidptosis. Salazosulfapyridine, Sorafenib, and HG106 inhibit SLC7A11 and induce intracellular ROS accumulation and ferroptosis to suppress tumor progression. Bay-876, KL-11743, STF-31 and Glutor promote disulfidptosis by inhibiting GLUT. This figure was created with Figdraw.

Many studies have focused on the direct inhibition of SLC7A11 transporter activity, such as the SLC7A11 inhibitors salazosulfapyridine, sorafenib, HG106, and bosutinib [127]. Salazosulfapyridine exerts its anticancer effect by blocking prostaglandin synthesis, whereas sorafenib and bosutinib act as multikinase inhibitors in oncology treatment. Additionally, natural compounds such as curculigoside and glabridin downregulate SLC7A11 expression, reducing cystine import and GSH synthesis to promote ferroptosis and disulfidptosis. HG106 has recently been identified as an SLC7A11 inhibitor [94], but its specific mechanism is not yet completely clear. In conclusion, despite the existence of SLC7A11 inhibitors for the treatment of tumors, there is still a need for further identification of effective and specific SLC7A11 inhibitors, and there is still a need to study their relevant mechanisms of action, conduct relevant clinical trials, and ultimately use them in tumor therapy (Fig. 6).

In addition to the study of SLC7A11 inhibitors, some researchers have proposed the induction of disulfidptosis for tumor treatment. Liu et al. reported that glucose transporter inhibitors are more susceptible to disulfidptosis in highly expressed SLC7A11 tumor cells. SLC7A11 high-expressing tumor cells are specifically targeted for cell death by GLUT inhibitors. GLUT1 is a membrane protein consisting of 12 transmembrane helices and an intracellular structural domain that transports glucose in a concentration gradient.

In a study involving BAY-876 (a GLUT1 inhibitor) and KL-11743 (a GLUT1/3 inhibitor), the treatment of renal cell carcinoma mouse transplanted tumor cells with these inhibitors resulted in a decrease in glucose uptake [128]. This decrease in the intracellular glucose level led to a change in the NADP+/NADPH ratio, ultimately causing cell death. The efficacy of these inhibitors was further confirmed in a xenograft model with high SLC7A11 expression. The inhibition of GLUTs induced the formation of disulfide bonds in actin skeleton proteins, ultimately leading to cytoskeletal collapse and subsequent cell death. These findings suggest that GLUT inhibitors can mimic the effects of glucose starvation. Emerging natural products such as genistein and epigallocatechin gallate, which bind to GLUT1/3 and impair glucose transport, have shown promise in triggering disulfidptosis in SLC7A11-high tumors [129].

In addition to SLC7A11 and GLUT inhibitors, recent studies have highlighted PPARγ antagonists (GW9662 and T0070907) as dual regulators of ferroptosis and disulfidptosis [130]. These compounds upregulate HMOX1 (which promotes ferroptosis) and SLC7A11 (which induces disulfidptosis), synergistically inhibiting tumor growth while recruiting dendritic cells and CD8+ T cells to the tumor microenvironment. Furthermore, mitochondrial modulators such as R001 (inhibiting G6PD and TrxR1) and DTT/β-mercaptoethanol (reducing disulfide stress) offer additional strategies to manipulate disulfidptosis pathways (Table 7).

**Table 7:** Classification of metabolic and redox modulators as disulfidptosis inducers in antitumor therapy.

Drug class	Category	Mechanism of action	Drug list
SLC7A11 inhibitors	Metabolic Regulators	Directly inhibit transporter activity, reduce cystine uptake	Sulfasalazine, Sorafenib, HG106
GLUT inhibitors	Glucose Metabolism Inhibitors	Block glucose transport, induce metabolic vulnerability, trigger disulfide bond accumulation	BAY-876, KL-11743
PPARγ antagonists	Dual Regulators	Upregulate HMOX1 (ferroptosis) and SLC7A11 (disulfidptosis), synergistically inhibit tumors and activate immune microenvironment	GW9662, T0070907
Mitochondrial modulators	Redox Regulators	Inhibit G6PD/TrxR1 or reduce disulfide stress	R001, Dithiothreitol (DTT), β-mercaptoethanol

Although important discoveries have been made about disulfidptosis, the therapeutic efficacy of disulfidptosis and potential safety issues in humans still need attention.

In the treatment of tumors, the effectiveness of a single GLUT inhibitor is limited and heavily relies on the high expression of SLC7A11. Therefore, for the clinical use of GLUT inhibitors, identifying patients with tumors exhibiting high SLC7A11 expression is crucial. Finding a suitable and efficient assay for assessing SLC7A11 expression is essential for optimizing the therapeutic efficacy of GLUT inhibitors. Additionally, owing to the restricted therapeutic potential of a single GLUT inhibitor, the development of combination therapy with other drugs could improve treatment outcomes. While therapeutic studies on disulfiram have focused primarily on tumor cells, there has been notable oversight of noncancer stromal and immune cells. These cells play a significant role in tumor progression and maintenance, underscoring the importance of considering their involvement in treatment strategies.

Related studies have shown that the induction of disulfidptosis by the inhibition of GLUTs also has the potential to affect nontumor cells, particularly immune cells, which poses a challenge for therapeutic studies of disulfidptosis [95]. In addition, treatment with GLUT inhibitors may result in off-target effects, and inhibition of GLUT is not the only mechanism by which GLUT inhibitors suppress tumor growth. Finally, it remains to be determined whether GLUT inhibitors have the expected pharmacokinetic properties to advance to the clinic.

## Conclusions and perspectives

This review systematically delineates the pivotal role of disulfidptosis, a novel RCD modality, in tumor metabolic reprogramming. Unlike previous studies focused on single cancer types, we innovatively integrated pancancer datasets and single-cell transcriptomics to map the heterogeneous expression patterns of DRGs and their dynamic crosstalk with immune microenvironment remodeling. For example, GYS1 was experimentally validated as a novel oncogenic driver in triple-negative breast cancer through glycogen metabolism regulation, complementing the findings of Yao et al. on the lncRNA CASC8/c-Myc axis in pancreatic cancer. Furthermore, this study pioneers the development of a pancancer disulfidptosis activity score, revealing its correlation with immunosuppressive features (e.g., increased Treg infiltration and reduced CD8+ T cells), thereby providing a rationale for combining immune checkpoint blockade (anti-PD-1) as a synergistic strategy with Tang et al. TrxR1-targeted therapy in glioblastoma.

At the molecular level, SLC7A11-mediated cystine overload and NRF2/c-Myc-regulated pentose phosphate pathway activation was identified as core mechanisms. Unlike ferroptosis (GPX4-dependent) or cuproptosis (mitochondrial copper-driven), disulfidptosis triggers cytoskeletal collapse via aberrant disulfide crosslinking, yet its metabolic interaction with ferroptosis (e.g., GSH depletion) enables combination therapy. Shi et al. demonstrated that gaudichaudione H induces dual ferroptosis/disulfidptosis by stabilizing NRF2 to upregulate SLC7A11, highlighting the synergistic potential of natural compounds with targeted agents. However, limitations persist: most bioinformatics analyses lack functional validation of DRGs, and the regulatory roles of noncancer stromal cells and immune cells in disulfidptosis remain unexplored.

Future directions should prioritize deciphering crosstalk between disulfidptosis and other death pathways (e.g., necroptosis) via CRISPR-based screens to identify key nodes, akin to the CD8A-SLCO1B1 model in HCC by Chen et al.; developing precision therapies based on metabolic signatures, leveraging single-cell multiomics to optimize targeting strategies, such as combining GLUT inhibitors (BAY-876) with immunotherapy in SLC7A11-high tumors; and exploring synergies between natural compounds (e.g., genistein, epigallocatechin gallate) and existing therapies (e.g., PPARγ antagonists) to overcome metabolic heterogeneity in the TME. Through interdisciplinary collaboration and clinical translation, disulfidptosis research holds promise for redefining cancer therapeutics.
